# Analysis of trends of malaria from 2010 to 2017 in Boricha District, Southern Ethiopia

**DOI:** 10.1186/s12936-020-03169-w

**Published:** 2020-02-24

**Authors:** Desalegn Dabaro, Zewdie Birhanu, Delenasaw Yewhalaw

**Affiliations:** 1 Yirgalem Hospital Medical College, Yirgalem, Ethiopia; 2 Department of Medical Laboratory Sciences and Pathology, College of Health Sciences, Jimma University, Jimma, Ethiopia; 3 Department of Health Education and Behavioral Sciences, Jimma University, Jimma, Ethiopia; 4 Tropical and Infectious Diseases Research Center, Jimma University, Jimma, Ethiopia

**Keywords:** Malaria, Positivity rate, Incidence rate, Ethiopia

## Abstract

**Background:**

Ethiopia has made a significant progress of malaria control. Currently, the country has adopted and is implementing the World Health Organization very ambitious, but achievable, malaria elimination plan through extensive efforts. The regular evaluation of its performance is vital for plausible improvement. Thus, the aim of this study was to determine the trends of malaria infection in Boricha district, Southern Ethiopia.

**Methods:**

A retrospective study was conducted in all health facilities of the district. All malaria cases registered during 2010 to 2017 were reviewed to determine the trends of malaria morbidity. EpiData 3.1 was used for data entry and data were analysed using SPSS version 20.0.

**Results:**

A total of 135,607 malaria suspects were diagnosed using microscopy and rapid diagnostic test over the last 8 years, of which 29,554 (21.8%) were confirmed positive cases. *Plasmodium falciparum, Plasmodium vivax* and mixed infections (both species) accounted for 56.3%, 38.4% and 5.2% of cases, respectively. Except in 2013 and 2014, *Plasmodium falciparum* was the dominant species over *P. vivax*. Of the total confirmed cases 51.6% were adults (≥ 15 years) followed by 24.5% of 5–14 years, and 23.9% of under 5 years. In general, malaria morbidity was significantly reduced over the last 8 years. The positivity rate declined from 54.6% to 5% during 2010 to 2017, and the case incidence rate per 1000 population at risk also declined from 18.9 to 2.2 during the same period. Malaria was reported in all months of the year, with peaks in November, followed by September and July. Malaria transmission has strong association with season (x^2^ = 303.955, df = 22, p < 0.0001).

**Conclusion:**

In general, a significant reduction of malaria morbidity was observed over the past 8 years. However, further investigation using advanced diagnostic tools is vital to determine the level of sub-microscopic infections to guide the elimination plan. In addition, eco-epidemiological analysis at fine-scale level is essential to devise area-specific interventions.

## Background

Malaria remains a worldwide public health problem causing a significant number of morbidity, mortality, and huge economic loss despite an intensive implementation of interventions. Every year, it affects millions of people worldwide, with remarkable regional variations; developing countries, mainly in Africa, bear the largest share of burden followed by Asia. In 2017 alone, there were an estimated 219 million cases and 435,000 deaths due to malaria worldwide, where 92% of cases and 93% deaths occurred in Africa [1, 2].

Ethiopia is one of the malaria endemic countries in the world with 60% of population at risk of infection. It has long been a serious public health threat causing a significant number of sickness and deaths each year, and one of the top ten causes of morbidity in 2018. The disease occurs throughout the year with the considerable seasonal and geographical variations across the country. The peaks time of transmission ranges from September to December, following the main rainy seasons (June to September), while the minor transmission occurs during April and May, following the February to March rains. *Plasmodium falciparum* and *Plasmodium vivax* are the two dominant parasites, contributing 60% and 40% morbidity, respectively [3, 4].

In 2018 alone, there were 904,495 cases and 213 deaths of malaria in Ethiopia. The highest morbidity, 724,996 (80.1%), was due to *P. falciparum,* while remaining 166,340 (18.4%) and 13,159 (1.5%) were due to *P. vivax* and mixed infection (*P. falciparum *+* P. vivax),* respectively. Malaria affects all segments of population while the children bear the greatest share of burden followed by pregnant women [5, 6].

Recognizing the burden of disease, Ethiopia has implemented different malaria prevention and control strategies with the aim of reducing malaria morbidity, mortality and economic loss. The interventions used include distribution of insecticide-treated bed nets, indoor residual spraying, drainage of stagnant water, improved healthcare utilization, provision of intermittent preventive therapy to pregnant women, and early diagnosis and prompt treatment, prevention and rapid management of malaria epidemics and surveillance of disease. The country has been applying free of charge provision of malaria prevention and control services. Currently, Ethiopia is implementing malaria elimination programme to end the disease by 2030 [7, 8].

The concerted application of malaria control strategies has resulted in noticeable achievements throughout the world, and Ethiopia is one of the countries where remarkable progress has been made. As of the 2018 global malaria report, the incidence rate of malaria declined by 18% from 2010 to 2017. Similarly, in the same period, the estimated number of cases dropped from 239 million to 219 million, and the number of deaths dropped from 607,000 to 435,000. Despite this, malaria still remains a public health problem worldwide [1, 2, 9]. The incidence, prevalence and mortality rate of the disease in Ethiopia followed a similar pattern [10–14].

The analysis of malaria morbidity trend in every malarious area helps to understand the dynamics of disease transmission. Such information is crucial to devise evidence-based and area-specific interventions. Hence, the aim of this study was to analyse the 8 years trends of malaria transmission in Boricha district, Southern Ethiopia.

## Methods

### Study area

**Fig. 1:**
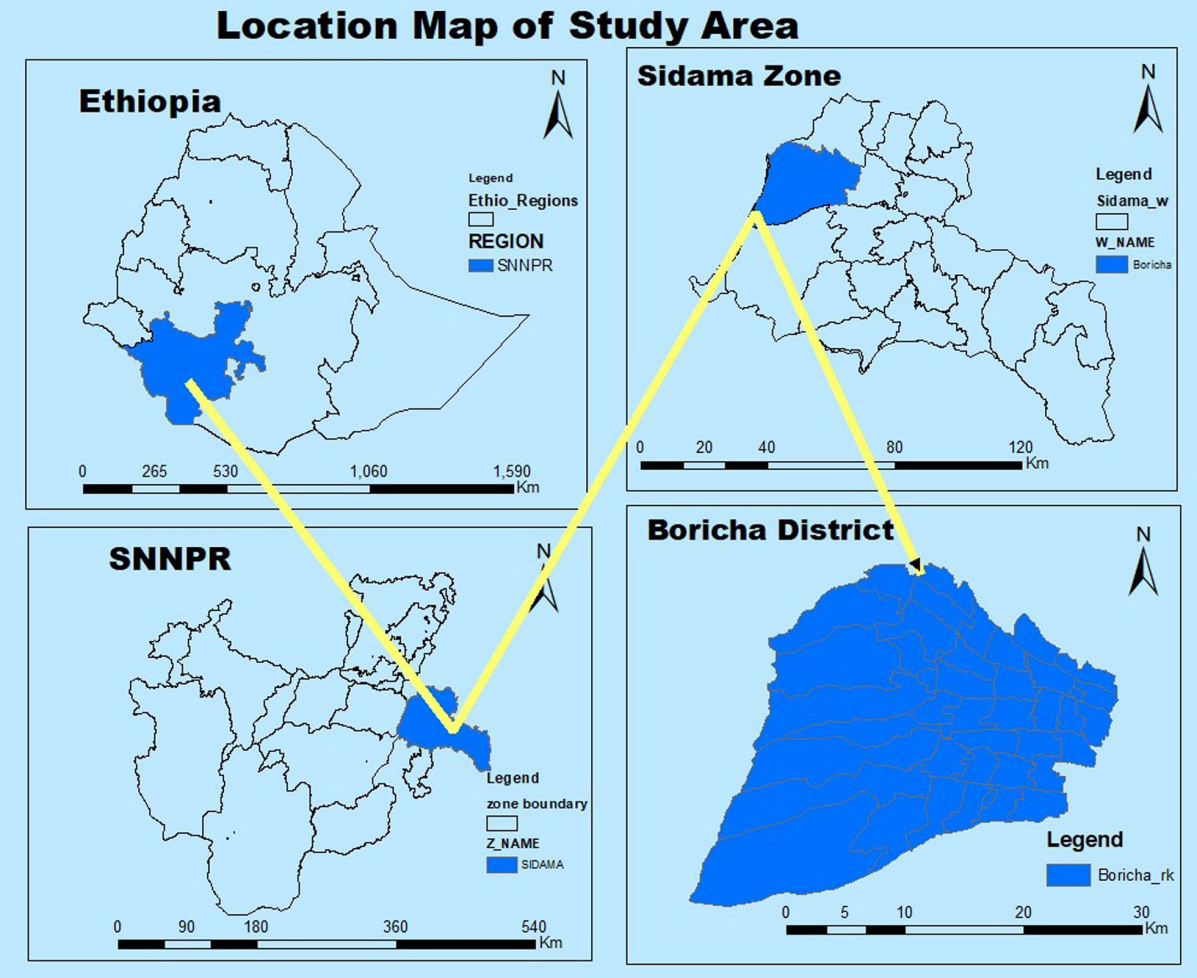
Map of study area

The study was conducted in Boricha district (Fig. 1), which is in the Sidama zone, Southern Ethiopia, located at 304 km from capital city of Ethiopia, Addis Ababa. It covers a total area of 588.1 km^2^. The altitude of the district ranges from 1001 to 2076 metres above sea level, with a mean annual rainfall ranging from 801 to 1000 millimetres and the mean annual temperature ranges from 17.6 to 22.5 °C [15].

At the last census, there were 325,161 populations in the district. Most of them live in rural areas, and agriculture is the major livelihood of the community. The common agricultural crops in the district where maize, teff, sweet potato and cabbage, while perennials includes banana, coffee, khat and sugarcane [16].

Administratively, the district has 39 rural and 3 urban *kebeles* (the lowest administrative unit). Both public and private health facilities provide healthcare service to the community. The public facilities include one primary hospital, ten health centres and 39 health posts. The private health facilities in the district include one non-governmental clinic, one medium clinic, five primary level clinics, three drug stores and eleven drug vendors [17].

A diagnosis and treatment service of malaria has been provided in all public health facilities and one private clinic (non-governmental clinic in the district). Diagnosis is either clinical or parasitological as per national guideline recommendation. A clinical diagnosis is based on signs and symptoms of patients, with a history of having fever in the last 48 h or if patients had gone to malaria-endemic areas within the last 30 days. Parasitological diagnosis is based on multi-species RDTs or light microscopy. Treatment using clinical diagnosis is the only option to diagnose malaria if there is no RDT and microscope. Artemether-lumefantrine and a single dose of primaquine is the recommended first-line treatment for confirmed *P. falciparum* or mixed infection (*P. falciparum* + *P. vivax),* and chloroquine plus 0.25 mg/kg primaquine for *P. vivax*. Oral quinine is recommended to first trimester pregnant women and underweighted children with *P. falciparum,* otherwise chloroquine is a safe treatment for pregnant women and infants. Intravenous or intramuscular artesunate is the preferred first-line treatment for severe malaria, and if this is not available intramuscular artemether is the alternative treatment. Intravenous quinine infusion is given when there is no artesunate or artemether [18].

### Study design

A health facility-based retrospective study was conducted to determine the trends of malaria morbidity over the 8 years (2010–2017) in Boricha district, Southern Ethiopia.

### Source of information

The source of information was malaria laboratory register logbook. All public health facilities (hospital, health centre and health posts and clinic) and one private clinic, the only private clinic that provides malaria diagnosis and treatment service were included in the study. All facilities use the same kind of malaria laboratory register, so that the variables captured were similar irrespective of type of health facilities.

### Data collection techniques

All laboratory registers of malaria morbidity were collected from all health facilities. Then the malaria morbidity data were transferred from the registers on the prepared Excel spreadsheet. The study variables included were date of diagnosis, address of patient, gender, age, tools used for diagnosis, result of diagnosis and species of parasite. The records which missed one or more of these variables were excluded from the study.

Prior to the study, data collectors and supervisor were trained for 2 days to insure the quality of data. They were trained on the data collection tools, variables of interest, rational, objective and significance of the study as well. Similarly, the data entry clerks were trained on the same points. The whole process, data capturing and data entry was daily supervised by principal investigator to ensure the completeness and consistency.

### Data analysis

Data were entered using EpiData 3.1 and analysed using Statistical Package for the Social Science (SPSS 20.0). Descriptive statistics was used to show the trends of malaria transmission in terms of seasons, years, gender, age and species of malaria parasite. Data were presented in percentage, tables and figures. The Pearson’s Chi square test was used to describe the associations of variables. Test of significance was estimated assuming $\alpha$ at 0.05 and a p-value less than 0.05 was considered significant. A seasonal index was analysed for each month to obtain the seasonal pattern of malaria transmission during the study period. A seasonal index is a measure of how a particular season compares with the average season. In this study, the season is a month. The malaria incidence rate per 1000 population at risk was computed using the total population of each year as a denominator and confirmed cases of malaria as a numerator.

### Operational definition

*A suspected malaria case* is a febrile illness with or without other symptoms that is suspected by a health worker as malaria infection.

*A confirmed malaria case* is confirmed cases of malaria using a diagnostic test (microscopy and rapid diagnostic test).

## Results

### Annual malaria case trends

A total of 135,607 malaria suspected cases were examined during 2010 to 2017, of these 29,554 (21.8%) were positive for malaria. Of the total suspected cases, 85,632 (63.1%) were diagnosed by microscope and the remaining 49,975 (36.9%) by RDT. The overall positivity rate was 24,168 (28.2%) and 5386 (10.8%), respectively.

**Fig. 2:**
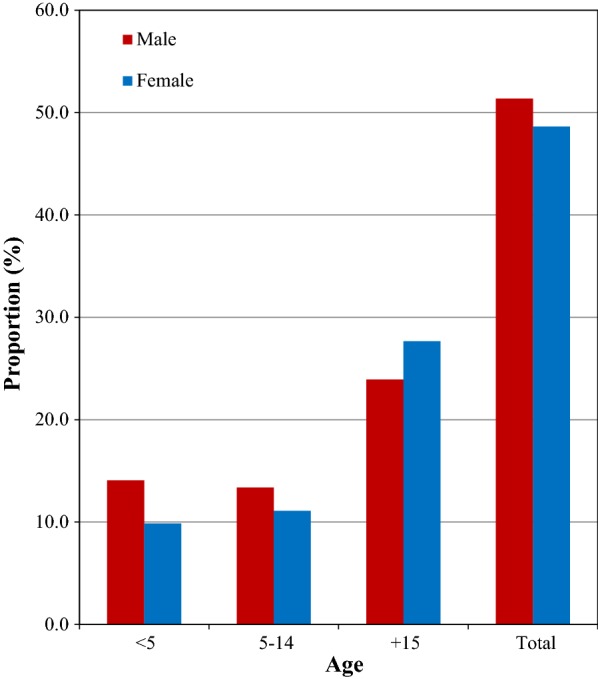
Proportion of malaria morbidity by age and sex in Boricha district, Southern Ethiopia (2010–2017)

Of all confirmed cases, 15,250 (51.6%) were adults of 15 years and above, and the remaining 7230 (24.5%) and 7074 (23.9%) were children of age 5–14 and < 5 years, respectively. Children < 15 years accounted for 48.4%. Regarding the gender distribution, 51.4% of overall confirmed cases were males and 48.6% were female. The proportion of childhood malaria was high in male than female, and the reverse was seen in adults. In general, malaria infection was significantly associated with age (x^2^ = 446.294, df = 6, p < 0.001) and gender (x^2^ = 348.001, df = 3, p < 0.001) (Fig. 2).

The number of malaria suspected cases were increased from 2010 to 2017 with the significant fluctuations from year to year. It was sharply raised during 2010 to 2013 and then declined gradually to 2017. Unlike suspected cases, the confirmed cases were declined over the last 8 years with considerable inter-annual variations. Generally, 87% reduction of malaria morbidity was observed in 2017 compared to 2010 (Table 1).

**Table 1:** Slide positivity rates and species composition of malaria parasite in Boricha District, Southern Ethiopia (2010–2017)

Year	Total febrile cases	Positivity rate n (%)				Malaria species n (%)		
		Total	*Pf*	*Pv*	Mixed	*Pf*	*Pv*	Mixed
2010	10,141	5538 (54.6)	4132 (40.7)	1255 (12.4)	151 (1.5)	4132 (74.6)	1255 (22.7)	151 (2.7)
2011	13,147	5528 (42.0)	3405 (25.9)	1620 (12.3)	503 (3.8)	3405 (61.6)	1620 (29.3)	503 (9.1)
2012	18,317	5134 (28.0)	2961 (16.2)	1968 (10.7)	205 (1.1)	2961 (57.7)	1968 (38.3)	205 (4.0)
2013	22,466	5093 (22.7)	2456 (10.9)	2567 (11.4)	70 (0.3)	2456 (48.2)	2567 (50.4)	70 (1.4)
2014	20,275	3642 (18.0)	1357 (6.7)	2123 (10.5)	162 (0.8)	1357 (37.3)	2123 (58.3)	162 (4.4)
2015	19,530	2353 (12.0)	1107 (5.7)	1011 (5.2)	235 (1.2)	1107 (47.0)	1011 (43.0)	235 (10.0)
2016	17,252	1549 (9.0)	823 (4.8)	559 (3.2)	167 (1.0)	823 (53.1)	559 (36.1)	167 (10.8)
2017	14,479	717 (5.0)	406 (2.8)	257 (1.8)	54 (0.4)	406 (56.6)	257 (35.8)	54 (7.5)
Total	135,607	29,554 (21.8)	16,647 (12.3)	11,360 (8.4)	1547 (1.1)	16,647 (56.3)	11,360 (38.4)	1547 (5.2)

Overall positivity rate was declined from 54.6% in 2010 to 5.0% in 2017. However, while insignificant variations of confirmed cases were observed during 2010 to 2013, the number of suspected cases during the same time period was sharply raised. Thus, the positivity rate was declined in the same time period, from 54.6 to 22.7%. The numbers of suspected and confirmed cases were declined relatively during 2014 and 2017 and so did for positivity rate (Table 1).

**Fig. 3:**
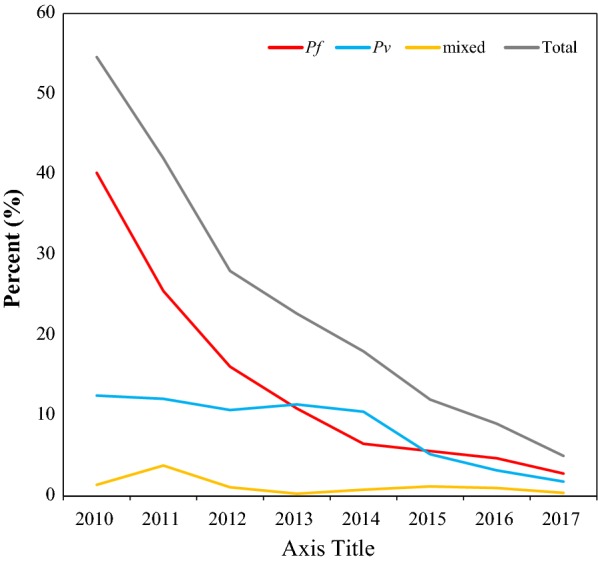
Malaria dynamics in Boricha district, Southern Ethiopia (2010–2017)

*Plasmodium falciparum* and *P. vivax* were the only species in study area, where *P. falciparum* accounted for 16,647 (56.3%) and *P. vivax* was 11,360 (38.4%), and the rest 1547 (5.2%) were mixed (*P. falciparum* +* P. vivax*) infection. *Plasmodium falciparum* was found to be the predominant species over *P. vivax,* except in 2013 and 2014 when *P. vivax* dominated. *Plasmodium falciparum* declined from 74.6 to 48.2% during 2010–2013, while *P. vivax* increased from 22.7% to 50.4% during the same period. However, from 2015 to 2017, *P. falciparum* again became the predominant species of the area (Table 1, Fig. 3).

**Fig. 4:**
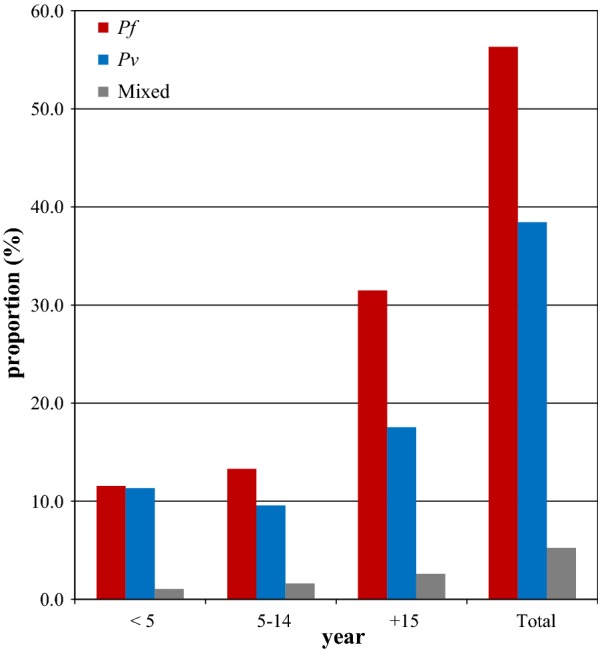
Distribution of *Plasmodium* species by age in Boricha district, Southern Ethiopia (2010–2017)

Regarding the distribution of parasite species in relation to age, children in the 5–14 years age group and adults (≥ 15 years) were most affected by *P. falciparum.* However, *P. falciparum* and *P. vivax* were evenly distributed in children < 5 years of age (Fig. 4).

### Malaria incidence rate

**Fig. 5:**
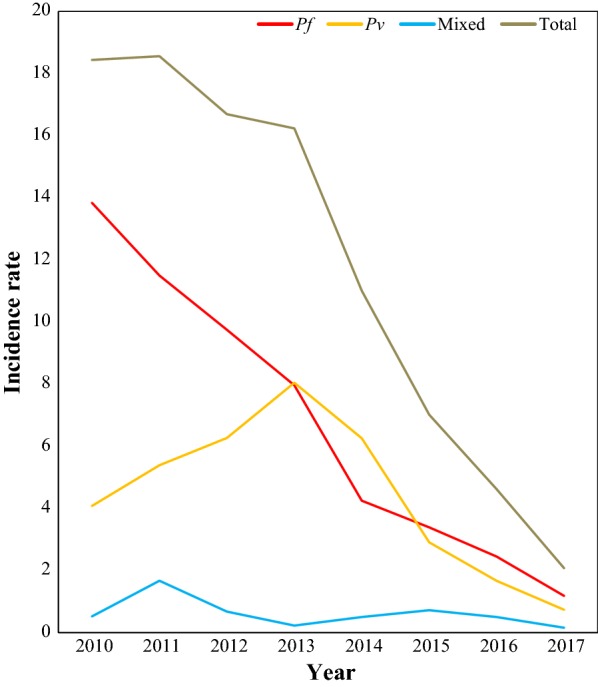
Malaria incidence rate per 1000 population at risk in Boricha district, Southern Ethiopia (2010–2017)

Overall malaria incidence was declined from 18.9 in 2010 to 2.2 in 2017 (mean = 12.12) per 1000 population at risk with a significant inter-annual variations. The incidence rate was high during the first 4 years, 2010 to 2013 (18.7–16.7 per 1000 population at risk). However, it was sharply declined from 11.4 in 2014 to 2.2 in 2017 per 1000 population at risk) (Fig. 5).

### Malaria incidence rate by age groups

**Fig. 6:**
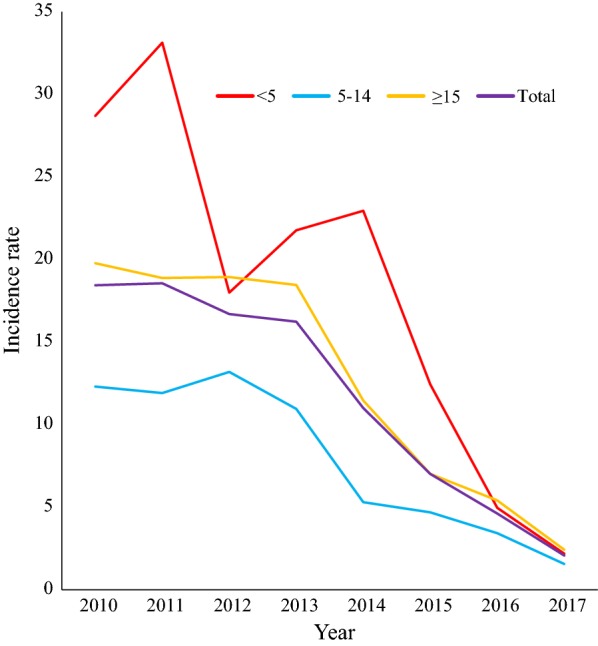
Incidence rate per 1000 population at risk by age group in Boricha district, Southern Ethiopia (2010–2017)

Remarkable variations of disease incidence were also observed in different age groups. It declined from 33.8 to 2.3 in children < 5 years of age, 13.5 to 1.7 in children 5–14 years of age and from 20.1 to 2.6 in adults ≥ 15 year age (Fig. 6).

### Seasonal variation of malaria cases

Table 2 displays the malaria cases from 2010 to 2017. The highest number of malaria cases was recorded in 2010 (5538 cases) while the lowest was in 2017 (717 cases). The highest standard deviation was seen in 2012, which implies that, the reported cases in that year had the highest monthly variations, whereas the lowest standard deviation was recorded in 2017, implying that the monthly reported cases did not vary significantly.

**Table 2:** Seasonal distribution of malaria infection in Boricha district, Southern Ethiopia

Year	Number of months	Mean	Standard deviation	Minimum	Maximum	Total malaria
2010	12	461.5	248.193	3	825	5538
2011	12	460.67	129.543	293	779	5528
2012	12	427.83	273.519	120	888	5134
2013	12	424.42	100.939	294	611	5093
2014	12	303.50	78.735	195	443	3642
2015	12	196.08	45.987	100	265	2353
2016	12	129.08	44.574	74	227	1549
2017	12	59.75	20.552	28	99	717
Total	96	307.85	207.409	3	888	29,554

**Fig. 7:**
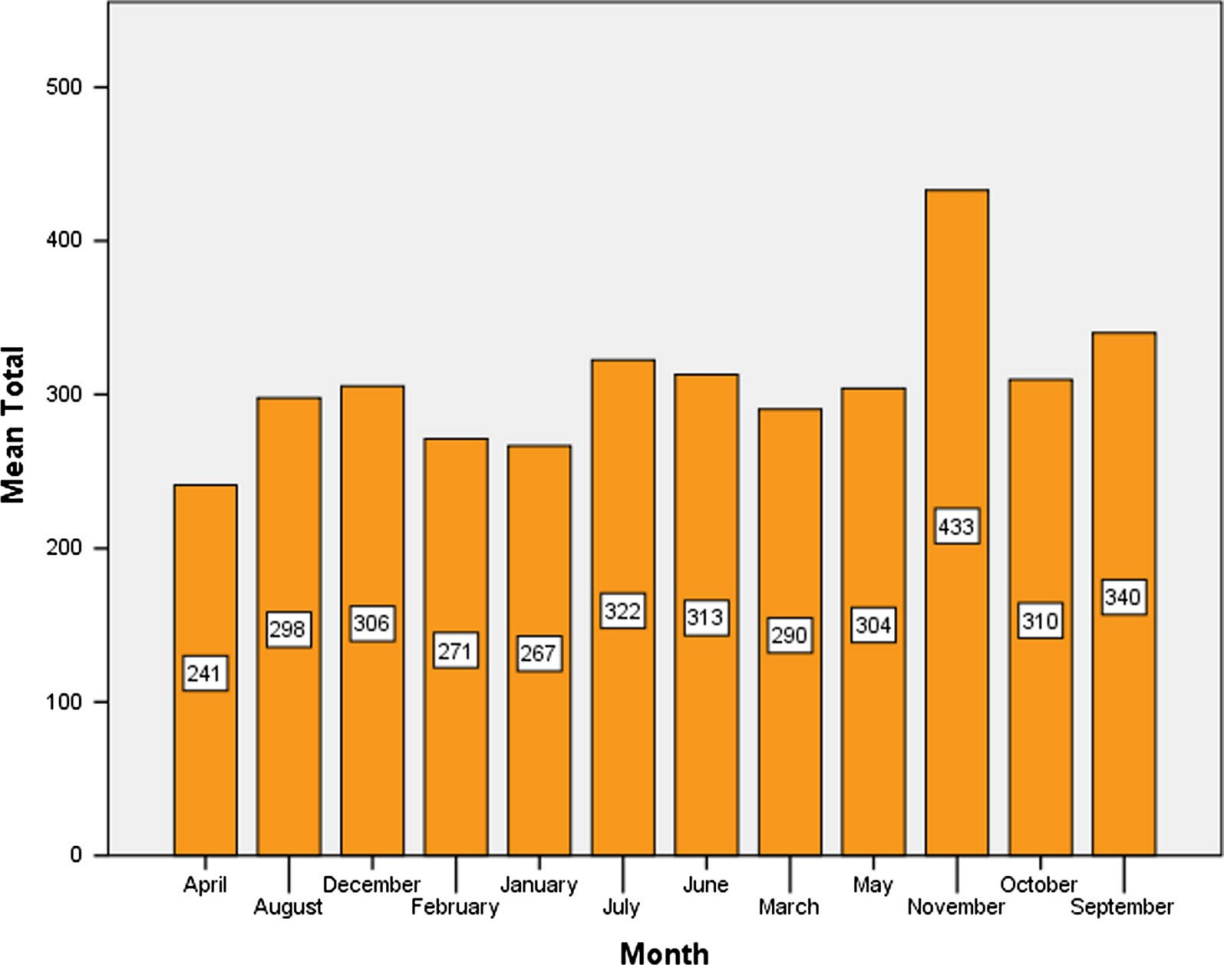
Seasonal indexes of 8 years malaria cases in Boricha district, Southern Ethiopia (2010–2017)

The monthly analysis of malaria cases consisted of 96 months. That is, 12 month each for the 8 year period. The seasonal indices are obtained and shown in Fig. 7. The seasonal indexes plot indicates that, malaria cases in September, October, November, May, June, and July are above the average malaria cases (307.85), whiles those of March, January, February, December, August and April are below the average. From this malaria transmission in the months of November, September and October had the highest transmission whilst April had the lowest. In addition, the result of Pearson’s Chi-square analysis showed statistically significant association of malaria transmission with the seasons (x^2^ = 325.349, df = 22, p < 0.0001).

**Fig. 8:**
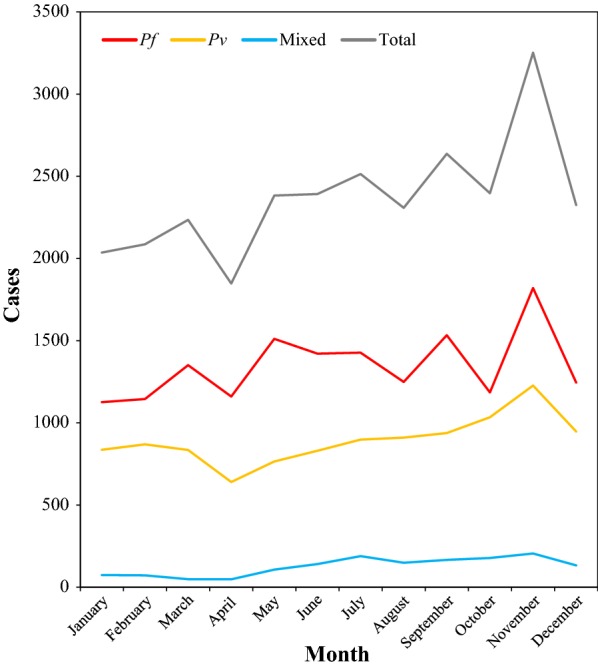
Seasonal variations of malaria transmission in Boricha District, Southern Ethiopia (2010–2017)

Both, *P. falciparum* and *P. vivax* were reported in all seasons during the study period*. Plasmodium falciparum* peaked in September, November and May where 30.1% of overall *P. falciparum* was reported. On the other hand, *P. vivax* peaked during October, November and December where 30.3% of total *P. vivax* was reported (Fig. 8).

## Discussion

The findings of the present study indicate a significant reduction of malaria morbidity over the past 8 years. The total caseload declined in the successive years with the significant inter-annual and intra-annual variations. In this particular study, overall malaria positivity rate was 21.8%. The declined malaria positivity was reported by similar studies from Northern Ethiopia and elsewhere, where the overall malaria positivity rate was 5.4%, 5% and 7.52%, respectively [19–21]. In contrast, higher overall malaria positivity rates (32.6% to 39.6%) were reported from similar studies conducted in Southwest and Northern Ethiopia [22–25]. The possible contributing factors for observed variation could be the poor quality of laboratory diagnosis, less community awareness, inequitable health workforce deployment, microclimate variation, expansion of development projects like dams or irrigation, and insecticide and drug resistance [3, 26–28].

The incidence rate was also computed to see a clear insight of the disease at the population level and the overall incidence declined from 18.9 to 2.2 per 1000 population at risk, more than eightfold lower than it was in 2010. This is the evident that the study area is at low transmission of disease as per the country national malaria risk stratification for 2014–2020, which categorizes area with < 5 malaria cases/1000 population at risk under low transmission strata [3].

Since 2005, Ethiopia has been implementing multiple interventions in integrated manner throughout the country, including the study area. The interventions include use of LLINs, IRS, prompt diagnosis and treatment with artemisinin-based combination therapy, intermittent preventive treatment in pregnancy and environmental management. In addition, the country has achieved the substantial expansion of primary health care services that is proven to improve the availability and accessibility of the healthcare services through facilitating early case detection and appropriate management. The country has also been intensifying disease control effort by implementing community based malaria diagnosis by assigning dedicated health extension workers at kebele/village level. On top of this, the concerted activities of social and behavioural change communication have been conducted to improve the community acceptance of existing malaria interventions. Such concerted efforts have also been applied in the study area and could be a possible justification for the observed decline of disease transmission at the area [6, 7, 12, 27].

While the overall malaria morbidly declined, the number of malaria suspects increased over the time. This could be due to the increased number of population at risk of contracting malaria in contrast to the dramatic reduction of disease. In addition, the improved availability and accessibility of health services coupled with the improved health-seeking behaviour could increase the health service utilization rate of the community and resulted in increased number of patients visiting health facilities. Thus, this could be the possible explanation for the increased number of reported malaria suspects in the area [12, 27].

In this study, the burden of malaria morbidity was more concentrated in older age groups than in children, accounting 51.6%. This is comparable with the findings from Raya Azebo district, Ataye district and Bahir Dar city in Northern Ethiopia [20, 29, 30]. In addition, even higher (73.7%) of malaria patients were adults of age fifteen and above was reported from Southwest Ethiopia [25]. The contributing factors for such higher burden of disease among adults could be due to their frequent engagement in different activities like agriculture, trade and other occupational risks that increase the exposure to infective mosquito bites.

Though the overall malaria burden was highest in adults (≥ 15 years of age), relatively the incidence of disease per 1000 population at risk was also high in children < 5 years during 2010–2011 and 2013–2014, and sharply declined in the latter years of the study. This indicates that age stratified incidence, which considers the respective population of each age group as a denominator provides the better understanding of trends in various age categories than using absolute number or the proportion of cases. The remarkable reduction of < 5 years malaria incidence rate could be attributed to extensive efforts of control intervention in targeting children < 5 years [7, 31].

In contrast, the incidence of malaria per 1000 population at risk was relatively low in 5–14 years children throughout the study period. This could be attributed to little attention of malaria treatment given to this particular age group. According to the study in Malawi, children in 5–14 years were less frequently brought to malaria treatment and they serve as reservoirs of malaria parasite and asymptomatic malaria was more common [32, 33]. This indicates that age-stratified submicroscopic malaria survey at community level is required for better understanding of malaria distribution in different age groups.

In this particular study, slightly more males were affected by malaria than females. This finding is comparable with studies from various localities in Ethiopia that reported higher malaria burden among males than females [20, 23, 25, 29, 34].

Regarding the species distribution, *P. falciparum* and *P. vivax* accounted for 56.3% and 38.4% of overall reported cases, and the rest was mixed infection. This finding is consistent with national figures and other similar studies in Ethiopia that reported dominance of *P. falciparum* over *P. vivax* [20, 29, 35]. *Plasmodium falciparum* was predominately reported throughout the study period except in 2013 and 2014 where a trend of disease transmission was shifted to *P. vivax*. The similar findings were reported from the Northwest and Southwest Ethiopia [22, 25, 29].

The persistent dominance of *P. falciparum* over *P. vivax* could be due to the severity of disease, drug resistance and gap of programme performance. On the other hand, the proportion of *P. vivax* was low except in 2 years of the study. The possible explanation for the observed dominance of *P. vivax* over *P. falciparum* could be due to the less attention of malaria programme to prevent *P. vivax* and less laboratory capacities to identify species [3, 36].

In this study, the secondary data were used to analyse malaria transmission trend, which could be the possible limitations of the study. The data collected from health facilities only and this might underestimate the actual burden of malaria in the community. Another problem of secondary data is the quality of report might sometime be not to the expected level. In addition, malaria mortality data were not recorded in the register and thus the trends of malaria mortality was not reported in this study. Therefore, interpretation of the findings should be with the precaution.

## Conclusion

In conclusion, malaria morbidity was significantly declined in the study area from 2010 to 2017. There was the considerable inter-annual and intra-annual variations of transmission, and inconsistent distribution of disease burden in different age groups and gender. Understanding the temporal and spatial distribution of disease is essential to plausible planning of interventions. In addition, further investigation using advanced diagnostics is vital to guide the elimination plan.

## Data Availability

All data underlying the findings are available from corresponding author on reasonable request.

## Abbreviations

SPSS: Statistical Package for the Social Science
IRS: Indoor residual spray
RDT: Rapid diagnostic test
*Pf*: *Plasmodium falciparum*
*Pv*: *Plasmodium vivax*
